# Hybrid Single-Packet IP Traceback with Low Storage and High Accuracy

**DOI:** 10.1155/2014/239280

**Published:** 2014-02-23

**Authors:** Ming Hour Yang

**Affiliations:** Department of Information and Computer Science, Chung Yuan Christian University, No. 200, Chung Pei Road, Chung Li City, Taoyuan County 32023, Taiwan

## Abstract

Traceback schemes have been proposed to trace the sources of attacks that usually hide by spoofing their IP addresses. Among these methods, schemes using packet logging can achieve single-packet traceback. But packet logging demands high storage on routers and therefore makes IP traceback impractical. For lower storage requirement, packet logging and packet marking are fused to make hybrid single-packet IP traceback. Despite such attempts, their storage still increases with packet numbers. That is why RIHT bounds its storage with path numbers to guarantee low storage. RIHT uses IP header's ID and offset fields to mark packets, so it inevitably suffers from fragment and drop issues for its packet reassembly. Although the 16-bit hybrid IP traceback schemes, for example, MORE, can mitigate the fragment problem, their storage requirement grows up with packet numbers. To solve the storage and fragment problems in one shot, we propose a single-packet IP traceback scheme that only uses packets' ID field for marking. Our major contributions are as follows: (1) our fragmented packets with tracing marks can be reassembled; (2) our storage is not affected by packet numbers; (3) it is the first hybrid single-packet IP traceback scheme to achieve zero false positive and zero false negative rates.

## 1. Introduction

With the rapid growth of the internet, various internet applications have been developed for different purposes. However, malicious users may launch distributed/denial of service (D/DoS) attacks to disrupt the service of a server. According to the number of attacking packets, D/DoS attacks can be categorized into flooding-based attacks and software exploit attacks [1]. In flooding-based attacks, adversaries would send huge amount of forged source packets to exhaust victim's limited resources. As for software exploit attacks, attackers need to find hosts' vulnerabilities and then launch attacks with only a few packets, for example, Teardrop attacks and LAND attacks. Since most edge routers do not check a packet's origin address, it is difficult for core routers to recognize each packet's source address. These source IP addresses can be spoofed when an attacker wants to evade tracing. Therefore, how to locate the real source of impersonation attacks has become an urgent issue today.

In order to trace the real source of flooding-based packets, packet-marking schemes use each packet's IP header to mark the packet's route. These schemes can be put into two categories, probabilistic packet marking (PPM) [2–7] and deterministic packet marking (DPM) [8–10]. Savage et al. propose a PPM scheme with edge sampling, which is called fragment marking scheme (FMS) [6]. However, collision of hashed pieces of routes can lead a FMS to the wrong origin of attacks. Hence, in order to lower the false positive rate and to reduce the computation load and time in path reconstruction, Song and Perrig introduce an advanced marking scheme [3], and Yaar et al. propose FIT [2]. In their schemes, they reduce the attack packets that are required for path reconstruction with the help of the known network topologies. Besides, Liu et al.'s dynamic probabilistic packet marking (DPPM) [5] and Paruchuri et al.'s TTL-based PPM (TPM) [7] determine the probability of marking according to the number of hops in a route. This further decreases the number of packets required in their path reconstruction. But since most marked routers in DPPM and TPM are near the victim, it turns out their schemes need lots of packets to reconstruct an attack path. To improve this part, Tian et al. propose an adaptive probabilistic marking scheme [4] in which every router on the same path has equal marking probability. Belenky and Ansari's DPM traceback schemes [8, 9] only demand few packets for path reconstruction. But, their schemes require full compliance of every border router, and they are unable to deal with attacks from multiple sources. For this reason, Belenky and Ansari soon come up with a hash-based DPM [10] to get around such a problem. But they need to collect at least eight packets to rebuild an attack path.

To trace the origins of software exploit attacks with only one packet, Snoeren et al. propose SPIE [11] to digest the unchanged parts of a packet and use a bloom filter [12] to log the digests. However, this scheme requires large storage and has false positives because their packet digests in each log table may have collision [11]. In order to lower the chance of collision, Zhang and Guan propose TOPO [13]. They try to use each upstream router's identifier to lower the false positive rate of SPIE. But this scheme still requires large storage for logging.

Because of the high storage requirement in logging-based schemes, hybrid single IP traceback methods [14–17] have been proposed. Packet marking and packet logging are fused in these schemes to reduce the storage requirement of routers. Despite their efforts, their storage still grows with packet numbers. It means the routers must refresh logged data when the accumulated packet digests exceed the quota on each router. Therefore, when an intrusion detection system (IDS) detects an attack and follows these schemes' tracking to a refreshed router's log, false negatives occur in their path reconstruction. To deal with the storage problem in MRT [17] and MORE [16] and to prevent collision in log tables, M.-H. Yang and M.-C. Yang propose RIHT [18]. Its storage requirement is bounded by path numbers and its simulations, implemented on CAIDA's topology data [19], show that it requires only 320 KB for packet logging. Therefore, RIHT does not need to refresh its routers' logged data; hence, no false negatives in its path reconstruction.

MRT and RIHT use each IP header's ID and fragment flags and fragment offset as their 32-bit marking fields. But the fragment flag is used to judge whether a packet has been fragmented or not. If its value is modified by traceback schemes, a receiving end is not able to judge fragmentation. Besides, when a marked packet's size exceeds a router's maximum transition unit (MTU), the packet will be fragmented. When a router supports IPsec, it may need to add ESP's header to each packet. This increases the length of a packet and the chance of fragmentation. In fact, John and Tafvelln [20] point out that 63% fragmented packets are ESP packets. With the high chance of fragmentation and modified values of the three fields, packet reassembly is difficult in the two schemes. Moreover, according to RFC 6274 [21], MRT's and RIHT's marked packets may be dropped. If the values written in their fragment offset are larger than the field's limit, then the packet will be dropped.

Despite the fact that current hybrid IP traceback schemes have been able to track single packet attacks and that RIHT has reduced the storage requirement to an extent that a router does not need to refresh its tracing logs, packet fragmentation and packet drop issues can still fail their path reconstruction. Therefore, we propose a new 16-bit hybrid single IP traceback scheme that uses only ID field of an IP header for our packet marking. Our major contributions include the following.

- Our proposed scheme is the first to solve both the storage and the fragment problems.
- Our scheme passes the packet fragmentation check in RFC 6274 because we do not need to overwrite fragment offset.
- We are able to reassemble fragmented packets before/after logging [8].
- Zero false positive and zero false negative.

In the following section, we survey related studies on Huffman codes, MRT, MORE, and RIHT schemes.  Section 3 details our traceback scheme. In Section 4, we run simulations to analyze the storage requirement and efficiency of path reconstruction in our scheme. We also compare it with existing hybrid IP traceback methods. Conclusion is drawn in Section 5.

## 2. Related Work

Hybrid single packet IP traceback schemes, such as Huffman codes, MRT, MORE, and RIHT, use routers' interface numbers, instead of node sampling or edge sampling, to mark a packet's route. Following a packet's route, these methods mark routers' interface numbers on the packet's IP header. However, marking space is not always enough for every router on a route. So, these methods integrate packet logging into their marking schemes by allowing a packet's mark to be temporarily stored on routers.

Since these schemes use interface numbers of routers for marking, they assume a router set *R* = {*R*_1_, *R*_2_, …, *R*_*i*_, …, *R*_*y*_} comprising *y* routers in a network and require all the *y* routers support these schemes. Also, they use the router's degree as a parameter in their marking schemes. The degree of a router is the number of its interfaces, but it does not include the ports connected to local networks. Here, we use *D*(*R*_*i*_) to denote router *R*_*i*_'s degree. Besides, these schemes need to maintain an interface table on each router in advance. The table keeps *R*_*i*_'s upstream interface numbers, which range from 0 to *D*(*R*_*i*_) − 1. We use UI_*i*_^*r*^ (or UI_*i*_ if there is no ambiguity) to denote *R*_*i*_'s upstream interface number on route *r*. In the following paragraphs, routes and paths will be used interchangeably.

In the marking process, each router has to put its UI_*i*_ into the marking field. Usually the easiest way is to encode UI_*i*_ with fixed-length coding. However, such approach does not use a packet's marking field efficiently if *D*(*R*_*i*_) is not a power of two. Choi and Dai [15] propose a marking scheme using Huffman coding to reduce the bits required for marking on a packet. It encodes UI_*i*_ by Huffman coding according to the traffic of each interface. Their analysis shows their scheme has better performance when the traffic distribution for each interface is unequal. Malliga and Tamilarasi propose MRT [17], which uses a 32-bit marking field and Modulo/Reverse modulo Technique. They use mathematical methods to mark the marking fields. In their marking scheme, the new marking field = marking field × *D*(*R*_*i*_) + UI_*i*_, which is computed by the routers to which a packet is forwarded. In their path reconstruction, the old marking field = marking field ÷ *D*(*R*_*i*_), which is computed by the routers to which a packet is traced back. The upstream interface number UI_*i*_ = marking field % *D*(*R*_*i*_). In the calculation, “%” is the modulo operation. When the old marking field <*D*(*R*_*i*_), they get the logged mark from the router. And the reconstruction process is repeated.

According to the analysis in RIHT, if MRT's marking field, after logging, is still 0 on the adjacent downstream router, the router will be identified as a logged one during traceback. As a result, it cannot find correct information on the router and is unable to find the origin of an attack. To prevent such a problem when UI_*i*_ = 0, RIHT modifies the formula of marking as new marking field = marking field  × (*D*(*R*_*i*_) + 1) + UI_*i*_ + 1. In path reconstruction, the old marking field = marking field ÷ (*D*(*R*_*i*_) + 1). The upstream interface number UI_*i*_ = marking field % (*D*(*R*_*i*_) + 1) − 1. They also lower RIHT's storage requirement for logging to about 320 KB. As RIHT's log table does not need to be refreshed, it effectively reduces the false negative rate.

**Figure 1:**
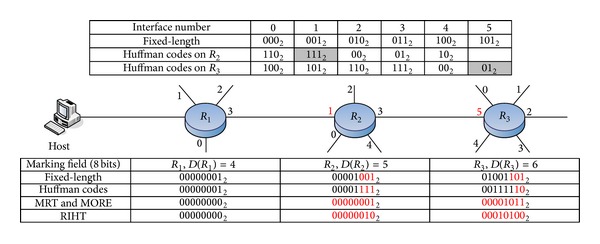
Example of traceback schemes that mark router interfaces.

Figure 1 illustrates the marking process of each traceback scheme which marks interface numbers of routers. Suppose that a packet is delivered from Host to *R*_1_, *R*_2_, and then *R*_3_ sequentially. The marking field is initialized on *R*_1_ and then marked on *R*_2_ and *R*_3_. As we can see in Figure 1, *R*_2_ receives *R*_1_'s packets from the upstream interface number 1 and *R*_3_ receives *R*_2_'s packets from the upstream interface number 5. In Huffman codes, *R*_2_, and *R*_3_ encode the interface numbers 1 and 5 as 111_2_ and 01_2_, respectively (see the grey cells in Figure 1). Reversals of codewords, that is, 111_2_ and 10_2,_ are appended into the marking field. In path reconstruction, *R*_2_ and *R*_3_ search the reversals of codewords to find the upstream routers. As RIHT has modified MRT, *R*_2_ computes the new marking field = 0 × 5 + 1 = 1(00000001_2_). And *R*_3_ computes the new marking field = 1 × 6 + 5 = 11(00001011_2_). In path reconstruction, *R*_3_ computes the upstream interface number = 11(00001011_2_) % 6 = 5, and the old marking field is 11/6 = 1(00000001_2_). *R*_2_ computes the upstream interface number = 1(00000001_2_) % 5 = 1 and the old marking field is 1 ÷ 5 = 0(00000000_2_). As for RIHT, *R*_2_ computes the new marking field = 0 × (5 + 1) + 1 + 1 = 2(00000010_2_). And *R*_3_ computes the new marking field = 2 × (6 + 1) + 5 + 1 = 20(00010100_2_). In path reconstruction, *R*_3_ computes the upstream interface number = 20(00010100_2_) % (6 + 1) – 1 = 5, and the old marking field is 20/(6 + 1) = 2(00000010_2_). *R*_2_ computes the upstream interface number = 2(00000010_2_) % (5 + 1) – 1 = 1 and the old marking field is 2 ÷ 5 = 0(00000000_2_).

As mentioned above, since MRT and RIHT use ID and fragment offset for packet marking, they have difficulty in reassembling fragmented packets. When the value marked in fragment offset is larger than the value defined in RFC 6274, the packet will be dropped by the routers. For these reasons, Malliga et al. propose a 16-bit hybrid traceback scheme called MORE, which only uses the 16-bit ID field for marking. Its logging and path reconstruction are identical to those in MRT. MORE turns the single log table into one table for each interface of a router. Such a change gives MORE smaller log tables and consequently prevents the insufficient marking space in a packet. But, since the scheme inherits MRT's logging method, it is still possible for its marking field to be 0 on the adjacent downstream router after logging. Then, the downstream router will be mistaken as a logged one and therefore lead their traceback to a wrong origin. Besides, like MRT and MORE, their storage requirements increase with packet numbers. It means when accumulated packet digests are larger than the quota of a router, especially when under flooding-based attacks, the router will refresh its logged data. Hence their path reconstruction fails [18].

## 3. A 16-Bit Hybrid Single Packet Traceback Scheme

In order to prevent packet fragmentation and insufficient storage for log tables, we propose a new hybrid IP traceback scheme that only uses the 16-bit ID field of an IP header; see Table 1. Further, our proposed marking scheme is able to pass the fragmentation check of RFC 6274.

**Figure 2:**
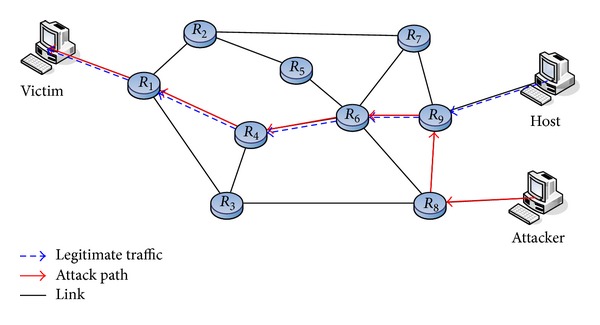
Network topology.

The topology of our scheme is illustrated in Figure 2. A router can be connected to a local network or other routers, or even both. A border router receives packets from its local network. A core router receives packets from other routers. For example, *R*_9_ serves as a border router when it receives packets from Host. However, it becomes a core router when receiving packets from *R*_8_.

Here, we assume that any router *R*_*i*_ has to satisfy the following assumptions.

- *R*_*i*_ is secure from attacks.
- A router creates an interface table and numbers the upstream interfaces from 0 to *D*(*R*_*i*_) − 1 in advance.
- A router knows whether a packet comes from a router or from a local network.
- This traceback scheme is viable on every router.

The notations used in our scheme are listed in Notation Section.

Our traceback scheme consists of two parts. The first includes marking/logging. The second deals with path reconstruction. The following subsections will detail the steps of our scheme.

### 3.1. Marking and Logging

When a border router receives a packet from its local network, it sets the packet's marking field as zero and forwards the packet to the next core router. Therefore, when adversaries send attack packets with a forged path in the marking field trying to confuse our tracking, we can still locate their origin correctly. On the other hand, when a core router *R*_*i*_ receives a packet *P*_*j*_, *R*_*i*_ uses packet *P*_*j*_'s mark, *P*_*j*_.mark, the incoming interface UI_*i*_, and the degree *D*(*R*_*i*_) to compute a new marking field mark_new_ = *P*_*j*_.mark × (*D*(*R*_*i*_) + 1) + UI_*i*_ + 1. If mark_new_ does not overflow, the core router *R*_*i*_ overwrites *P*_*j*_.mark with mark_new_ and then forwards the packet to the next router. If mark_new_ overflows, the core router *R*_*i*_ has to compute *H*(*P*_*j*_.mark) and insert *P*_*j*_.mark and UI_*i*_ as a pair into a log table.

Since the index of a single table is inevitably too long for 16-bit marking fields, we use multitables to store packets' logs. Therefore, we need to determine which table to store first. As shown in Algorithm 1, we compute hash value of the source IP of the packet *H*_tab_(*P*_*j*_.srcIP) to choose a log table *k*. Also, we hash packet *P*_*j*_'s mark to determine its index *l* = *H*_idx_(*P*_*j*_.mark). Then, we insert *P*_*j*_.mark and UI_*i*_ as a pair into the *l*th entry of table *k*, that is, *HT*_*k*_^*l*^. According to the value of *HT*_*k*_^*l*^, we have come to two situations: the indexed entry is either empty or occupied.

*Case  1*. If the indexed entry *HT*_*k*_^*l*^ is null, *R*_*i*_ writes *P*_*j*_.mark and UI_*i*_ in *HT*_*k*_^*l*^, as shown in Table 2.

*Case  2*. If *HT*_*k*_^*l*^ is not empty, we compare the packet's mark *P*_*j*_.mark and interface number UI_*i*_ with the logged value in *HT*_*k*_^*l*^.

*Case  2.1*. If the value in *HT*_*k*_^*l*^ matches the current packet's marking, it means the two packets have an identical route. So, *R*_*i*_ does not need to log this packet.

**Figure 3:**
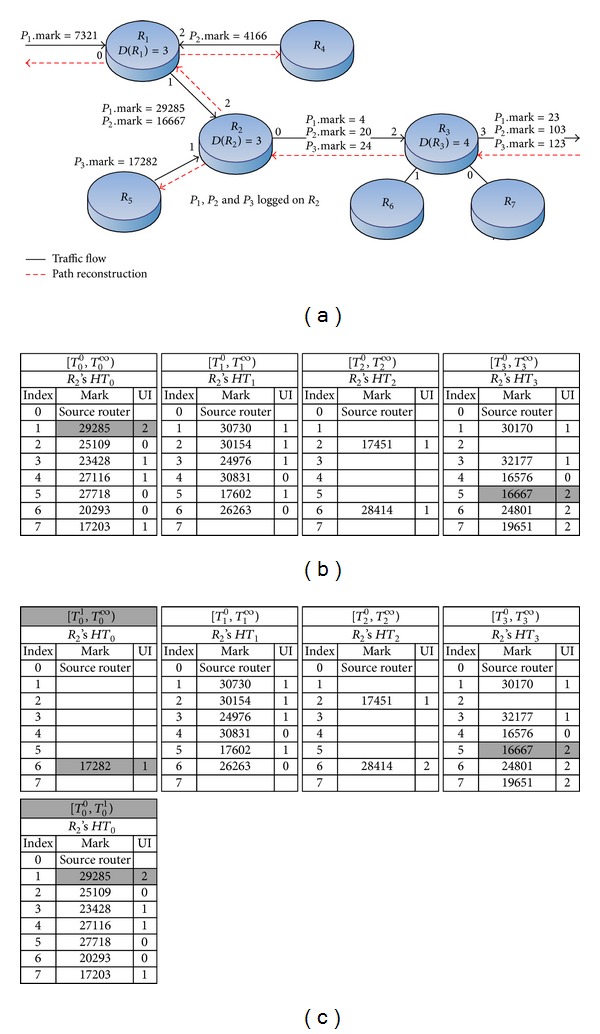
(a) Traffic flow of packets *P*_1_, *P*_2_, and *P*_3_. (b) Router *R*_2_'s log tables. (c) Generating a new *HT*_0_ when *R*_2_'s *HT*_0_ is full.

*Case  2.2*. If the two do not match, it means collision of *H*_idx_(*P*_*j*_.mark). Hence, we use the quadratic probing algorithm [22] to search *P*.mark and UI_*i*_ in *HT*_*k*_. If *P*.mark and UI_*i*_ are not found there, the core router inserts them as a pair into the table; see Algorithm 1. We use packet *P*_2_ and log table *HT*_3_ in Figure 3(b) to exemplify our logging scheme when collision occurs.

Next, we use the index *l* to compute a new mark mark_new_ = *l* × (*D*(*R*_*i*_) + 1) and overwrite the packet's *P*_*j*_.mark with the new mark. Then, the marked packet is forwarded to the next router.

Figure 3 exemplifies how router *R*_2_ logs three packets *P*_1_, *P*_2_, and *P*_3_, which have different upstream paths. The grey cells in Figure 3 show that the contents of *R*_2_'s log tables are modified after logging. When *R*_1_ receives a packet *P*_1_ whose mark is 7321, that is, *P*_1_.mark = 7321, *P*_1_ enters *R*_1_ from the interface 0; hence, UI_1_ = 0. According to our marking scheme, mark_new_ = 7321 × (3 + 1)+(0 + 1) = 29285. Since the new mark is within 65535, the maximum size of a 16-bit field, *R*_1_ rewrites *P*_1_'s mark *P*_1_.mark into mark_new_ and forwards the packet to the next router *R*_2_. After receiving *P*_1_ from the interface 2(UI_2_ = 2), *R*_2_ computes a new mark for *P*_1_, mark_new_ = 117143. Because the new mark is larger than 65535, *R*_2_ has to log the mark. First, it hashes the packet's source IP to get the table number *k* = *H*_tab_(*P*_1_.srcIP) = 0, so the new mark will be logged into the log table *HT*_0_. Then, it computes the table's index *l* = *H*_idx_(*P*_1_.mark) = 1. As *HT*_0_^1^ is null, *R*_2_ logs *P*_1_.mark and UI_2_ into *HT*_0_^1^; see the grey cell of table *HT*_0_ in Figure 3(b). Last, it uses the entry's index *l* to compute a new mark: mark_new_ = 1 × (3 + 1) = 4. It overwrites *P*_1_.mark with mark_new_ and forwards the packet to *R*_3_.

Figure 3(b) also helps to exemplify how we log a packet's mark if there is collision in a log table. When *P*_2_ arrives at router *R*_2_'s interface 2(UI_2_ = 2), *R*_2_ computes a new mark for *P*_2_, that is, mark_new_ = 66671. Because 66671 is larger than 65535, *R*_2_ computes *k* = *H*_tab_(*P*_2_.srcIP) = 3 and *l* = *H*_idx_(*P*_2_.mark) = 6. Since *HT*_3_^6^ is not empty and the value of *HT*_3_^6^ is different from *P*_2_.mark, we have to find another entry for logging in table *HT*_3_. Here, we use quadric probing algorithm to find a new entry that is available for logging. Then, we find the new entry's index *l* = (6 + 0.5 × 5 + 0.5 × 5^2^)%  8 = 5. Hence, *R*_2_ inserts *P*_2_.mark and UI_2_ as a pair into *HT*_3_^5^; see the grey cells of *HT*_3_ in Figure 3(b).

Last, we use Figure 3(c) as an example to show how we insert a mark into a log table when the table is full. Because we hash a packet's source IP to choose a log table, we do not balance the logging load of each table. Instead, we create our log tables in a two-dimensional way. All log tables are in one dimension. If a table is filled up, we create a new one and put the old one in another dimension. As shown in Figure 3(c), at first, all tables' created times are *T*_*k*_^0^ on the same horizon; here, *k* ranges from 0 to 3. When *HT*_0_ becomes full and we still need to log new data into it, router *R*_2_ modifies *HT*_0_'s time field as [*T*_0_^0^,  *T*_0_^1^). Then, *R*_2_ creates a new *HT*_0_ and set its time field as [*T*_0_^1^,  *T*_0_^*∞*^). The old table is placed below the new one, in a vertical direction. When *P*_3_ arrives *R*_2_ from the interface 1 (UI_2_ = 1), *R*_2_ computes a new mark for *P*_3_: mark_new_ = 69130. As the new mark is larger than 65535, *R*_2_ computes *k* = *H*_tab_(*P*_3_.srcIP) = 0. But the log table *HT*_0_ has been filled up, so *R*_2_ set current time *T*_0_^1^ on the table's time field to indicate its filled-up time, [*T*_0_^0^, *T*_0_^1^). Meanwhile, *R*_2_ creates a new table for *HT*_0_ and writes the current time *T*_0_^1^ to the table's time field to indicate its created time, [*T*_0_^1^, *T*_0_^*∞*^) see the first table and the one below it in Figure 3(c). At last, *R*_2_ computes *l* = *H*_idx_(*P*_3_.mark) = 1 and inserts *P*_3_.mark and UI_2_ into *HT*_0_^1^.

### 3.2. Path Reconstruction

When a victim detects an attacking packet *P*_*j*_, it sends to the upstream router a path reconstruction request, which includes the packet *P*_*j*_'s mark *P*_*j*_.mark, the packet's source address *P*_*j*_.srcIP and the packet's received time *T*_*j*_. After a router receives the request, it uses *P*_*j*_.mark to determine the incoming interface UI_*i*_ of packet *P*_*j*_. According to value of UI_*i*_, there are two situations.

*Case  1*. If UI_*i*_ = −1, it means the mark of *P*_*j*_ has been logged on this router. Then, the router hashes *P*_*j*_.srcIP to find out the log table that contains *P*_*j*_'s mark, that is, *k* = *H*_tab_(*P*_*j*_.srcIP). Because the router may have more than one table for *HT*_*k*_, we need to find out the one whose time field covers *P*_*j*_'s received time: *T*_*k*_^*s*^ < *T*_*j*_ < *T*_*k*_^*f*^. We then use *P*_*j*_.mark to compute the table's index *l* = *P*_*j*_.mark/(*D*(*R*_*i*_) + 1). If *l* = 0, it means this router is the source router. Otherwise, it gets mark_old_ and UI_*i*_ from *HT*_*k*_^*j*^ and overwrites the *P*_*j*_.mark with mark_old_. Last, it continues to trace the origin and sends the reconstruction request along with the *P*_*j*_.mark to its UI_*i*_'s upstream router. Detailed algorithm of our path reconstruction is shown in Algorithm 2.

*Case  2*. If UI_*i*_ ≠ −1, the requested router computes new mark_old_ and UI_*i*_ and overwrites *P*_*j*_.mark with mark_old_. Then, it sends the reconstruction request along with the *P*_*j*_.mark to its UI_*i*_'s upstream router.

We use the dotted lines in Figure 3 to exemplify how we reconstruct *P*_1_'s path. In this case, when *R*_3_ receives the reconstruction request that contains *P*_1_.mark = 23, *P*_1_.srcIP and *T*_*j*_, where *T*_0_ < *T*_*j*_ < *T*_1_, *R*_3_ computes the incoming interface number of *P*_1_. That is, UI_3_ = (23%  (4 + 1)) − 1 = 2. Since UI_3_ ≠ −1, it means *P*_1_ has not been logged on this router. *R*_3_ computes mark_old_ = 23/5 = 4 and overwrites *P*_1_.mark with mark_old_, that is, 4. Then, *R*_3_ sends a path reconstruction request with the new mark *P*_1_.mark through its interface 2 to the upstream router *R*_2_.

When *R*_2_ receives the request, it uses *P*_1_.mark to compute UI_2_ = (4%  (3 + 1)) − 1 = −1. Because UI_2_ = −1, it means *P*_1_ has been logged on *R*_2_. Next, *R*_2_ computes *k* = *H*_tab_(*P*_1_.srcIP) = 0 and *l* = 4/(3 + 1) = 1. Therefore, we know *P*_1_'s mark was logged in *R*_2_'s table HT_0_^1^. Furthermore, *R*_2_ finds out the table of *HT*_0_ whose time field [*T*_0_^0^, *T*_0_^1^) satisfies the requirement that *T*_*j*_ is between *T*_0_^0^ and *T*_0_^1^. Next, we get mark_old_ = 29285 and UI_2_ = 2 from the table of HT_0_^1^ whose time field is [*T*_0_^0^, *T*_0_^1^). *R*_2_ overwrites *P*_1_.mark with mark_old_, that is, 29285. Last *R*_2_ sends a path reconstruction request that contains *P*_1_.mark = 29285 through its interface 2 to the upstream router *R*_1_. The router *R*_1_ and following routers will repeat the steps mentioned above until the requested router's computation result is as follows: the index value is 0 and the interface number is −1, that is, the origin of the attack.

### 3.3. Reassembly of Packet Segments

According to the filter recommendation of RFC 6274 [21], a packet is fragmented when its size exceeds a router's maximum transmission unit (MTU). Because RIHT uses fragment offset field to mark packets, their offset value may be too large and exceed the maximum length of a packet during packet assembly. And the routers that comply with RFC 6274 will take the segment as abnormal and drop their marks. Furthermore, its 32-bit marking scheme uses ID, flags, and fragment offset fields for marking. This makes its packet reassembly at the destination almost impossible.

To prevent this problem, our method only uses each IP header's ID field for marking. It requires only 16 bits and prevents packet drop. In our scheme, any two arbitrary packets take the same path to a router if and only if they have the same marks on the same router. It means different packets on the same route will have the same ID because we use the field for marking. Although, according to Belenky and Ansari [8], the probability of fragment interlacing that results from out-of-order arrival of fragmented packets is 0.0018, it can still cause errors in packet reassembly. For this reason, we assemble all the segments according to their offset values. Then, we use the checksum to check the integrity of each packet and to filter those segments that have an identical offset value but belong to other packets. This allows us to verify whether the reassembly is correct. So, our scheme is able to reassemble most fragmented packets.

## 4. Performance Evaluation and Analysis

This section analyzes the storage requirement, precision, and computation loads of our traceback scheme. In the following paragraphs, we will first introduce our simulation environment. Then, we compare the performance of our scheme with that of other hybrid single-packet traceback schemes, that is, MRT, RIHT, and MORE. In the following simulations, the environment consists of a PC with Intel P4 930 3 GHz, 2 G RAM, and FreeBSD 6.2.

### 4.1. Simulation Environment

**Figure 4:**
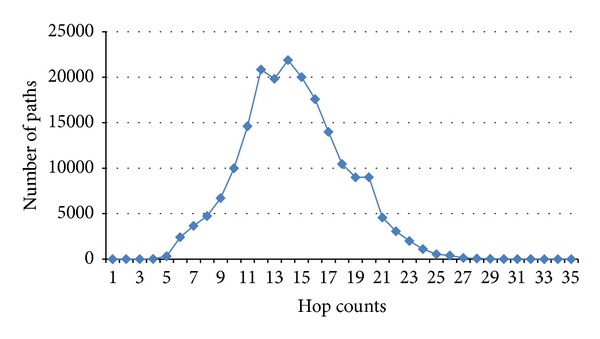
Distribution of path length.

To simulate the internet topology, we use the skitter project topology distributed by CAIDA [19] as our sample data set of the internet. The data set consists of paths to a specific host of the topology. We analyze CAIDA's skitter data and choose only 197,003 complete paths for our network topology. We ignore the incomplete paths in the data set which may cause routers not to respond to the ping command. The analysis results are illustrated in Figure 4. Total number of its routers are 130,267; its average hop count of paths is 14.42; and its average upstream degree is 2.63. There is a router whose degree is 434 and is the largest in the data set, while the second largest degree is only 157. The difference between the two degrees is 277. Therefore, according to CAIDA, the router whose degree is 434 requires the largest storage and our scheme will manage to meet its requirements.

### 4.2. Load Factor's Impact on Collision

Since collision may occur when we log packets' marks, we use the open addressing [22] method to deal with this problem. In the open addressing method, when a new entry has to be inserted, the slots are examined, starting with the hashed-to slot and proceeding in some probe sequence, until an unoccupied slot is found. When searching for an entry, the slots are scanned in the same sequence, until either the target record is found or an unused slot is found. Furthermore, to minimize the impact of collision on our scheme, we adopt the quadratic probing [22] as the probe sequence. Quadratic probing requires only light computation and is proved effective when we try to avoid clustering problem.

When we deal with a collision problem, we have to take into consideration the log table's load factor *α*, which is the proportion of logged paths to the log table's size. This factor can directly affect the number of collision. However, the calculation results of collision times may vary because we have two situations here, successful search and unsuccessful search. Unsuccessful search means that an entry has not been logged in a log table and therefore is to be inserted into an empty slot. A probe is performed each time collision occurs. The expected number of probes in unsuccessful search using open addressing is at most 1/(1 − *α*), assuming uniform hashing. Successful search means an entry has been logged in a hash table. The expected number of probes in successful search using open addressing is at most 1/*α*In⁡(1/(1 − *α*)), assuming uniform hashing.

**Figure 5:**
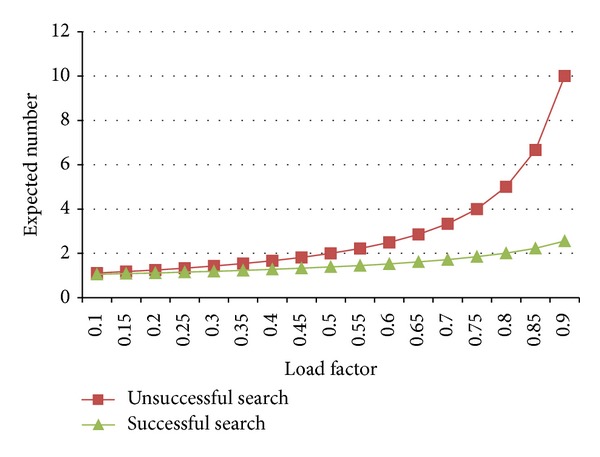
Expected number of probes.

The expected numbers of probes in the two situations are illustrated in Figure 5. We can see that if the load factor *α* is ≤0.5, the expected numbers of probes in the two situations are both 2 at most. Once *α* is >0.5, the collision in unsuccessful search drastically rises. Accordingly, we require that the load factor of each of our log table is 0.5 at most.

### 4.3. Analysis of Storage Requirement

Traceback schemes like MORE, RIHT, MRT, and ours need to log packets' marks on routers if their IP fields overflow. When a router has a larger degree, we will need more bits to encode it, which causes larger marks. And since larger marks lead to higher logging frequency, more storage is required for the downstream routers. In order to analyze the global storage requirements of current hybrid single-packet traceback schemes, we use the real Internet topology and require that each router have the same number of tables. But how many tables are required for our logging scheme? As there are totally 197,003 paths in the network topology, the number of a router's log tables *n* should satisfy (*n* − 1)×(*m* × *α* − 1) ≥ 197003 ≥ *n* × (*m* × *α* − 1). Each table only uses *m* × *α* − 1 entries to log packet marks. According to the logging scheme in RIHT [18], a router's log table with *m* entries is bounded by the number of upstream paths. Furthermore, *R*_*i*_ needs to find the entry index and computes mark_new_ = index × (*D*(*R*_*i*_) + 1). Then, it overwrites *P*_*j*_.mark with mark_new_. Therefore, we can say, the maximum entries of *R*_*i*_'s log table are *m* ≤ 65535/(*D*(*R*_*i*_) + 1). However, CAIDA's skitter data [19] points out that the number of paths of an upstream router may exceed 65535. Thus, a router needs multiple tables to log *P*_*j*_.mark. Besides, a large log table will also lead to a large mark *P*_*j*_.mark in packet *P*_*j*_. Consequently, the packet will have higher frequency of logging in the following routers, and the downstream routers will inevitably have larger storage loads. To prove this, we run the following simulations to analyze the storage requirements of MRT, MORE, RIHT, and the worst case of our scheme. Therefore, we send multiple packets to each of the 197,003 paths and then averaging all the routers' storage loads in logging. Among these schemes, both MORE and ours have to maintain a couple of log tables and an interface table on each router. Because the size of an interface table is relatively negligible, here we leave it out of our analysis.

**Figure 6:**
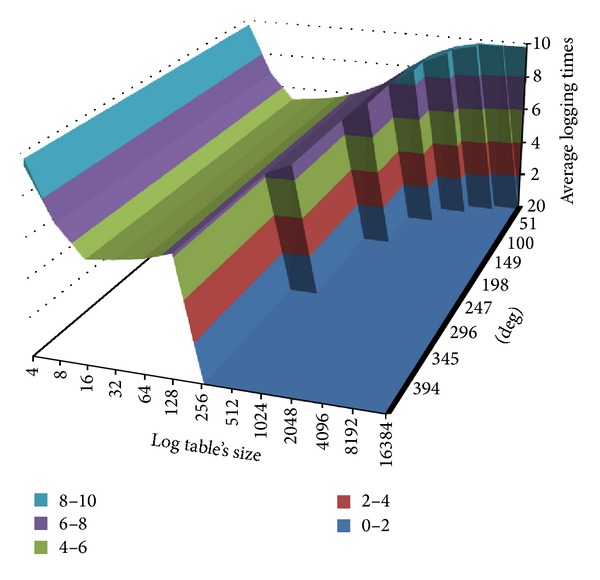
Relation among degrees, table size, and average logging times.

Figure 6 shows the relation among a router's interface numbers, its table size, and the average logging times of each router. As Figure 6 shows, the larger a router's degree is, the smaller a log table's maximum available entries are. Take the largest router in CAIDA's topology as an example. The router with a degree 409 may have a 128-entry log table at most. Also, when a log table's size is smaller than 16 entries there is a surge in average logging times. It is because a small log table can be filled up quickly and therefore results in the increase of log tables. That is why we set the minimum entries of all log tables as 16 in the following simulation.

**Figure 7:**
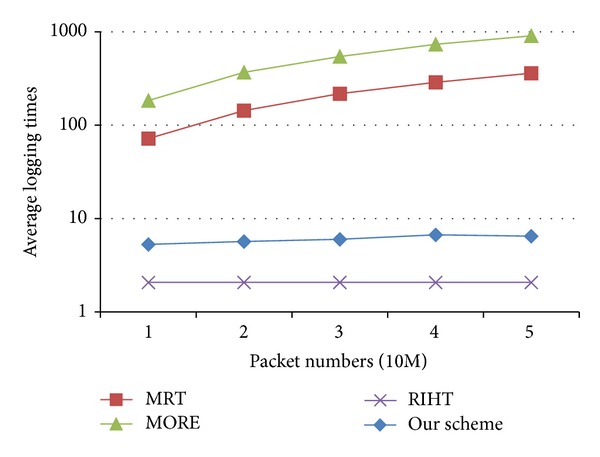
Comparison of logging times.

In Figure 7, we inject packets (from 10 million to 50 million) into the network to compare the logging times of our scheme with those of MRT, MORE, and RIHT. Because the logging times increase with packet numbers in MRT and MORE, their average logging times remain much higher than RIHT's and ours from the very beginning. Like RIHT, we bound our logging times with path numbers. The bounded logging times will not increase with packet numbers, so that we can keep our logging times low.

**Figure 8:**
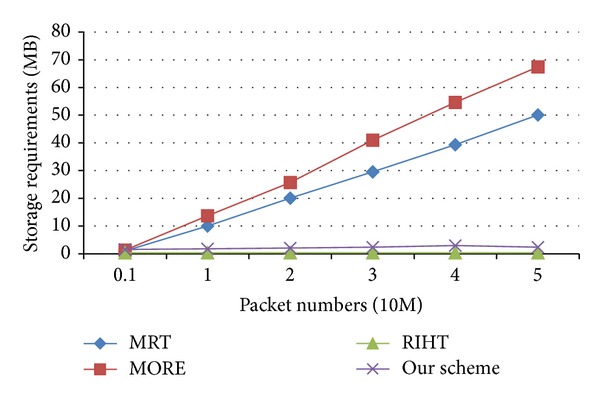
Comparison of storage requirements.

Figure 8 shows the storage requirements for MRT, MORE, RIHT, and our scheme on the largest router of CAIDA's topology. Each entry of MRT's log tables contains a 32-bit digest and a 32-bit marking field. In MORE, one entry contains a 32-bit digest and a 16-bit marking field. Thus, the storage requirements for their routers are *n* × 64 bits and *n* × 48 bits, respectively, where *n* is the number of logged packets on the router. On the same route, packets are logged on the same routers. When we inject packets (from 10 million to 50 million) into the network, the simulation results indicate that the storage requirement for MRT ranges from 0.99 MB to 50 MB; for MORE, from 1.28 MB to 67 MB; for our scheme about 2 MB; and for RIHT, 320 KB unchanged. For MRT and MORE, their storage requirements are lower than ours only when the packet numbers are below 1 million. However, a core router with 1000 gigabit bandwidth, or even wider, can receive much higher than 1 million packets shortly. If there is a flooding-based attack, the log tables in the two schemes will grow hugely in a short time. However, in RIHT and our scheme, the size of a hash table is fixed, which secures our scheme against flooding-based attacks.

RIHT's marking field is 32 bits, which is big enough for most marking. Therefore, it requires less logging and its storage is about 1.5 MB less than ours. But in our scheme, each router requires only 2 MB for storage. They will not need to drop logged marks for insufficient storage. So, our scheme is as practical as RIHT in storage requirement.

### 4.4. Analysis of Computation Loads

As for the computing time of a path reconstruction, both MRT and MORE require that a router uses the request packet's digest to find its previously stored marking field in the log table. However, their routers' log tables are unsorted, so they need an exhaustive search. Therefore, the average search time required for MRT is Θ(*n*), where *n* denotes the number of logged packets in a log table; and it is Θ(*n*_UI_*i*__) for MORE, where *n*_UI_*i*__ denotes the number of logged packets in the log table associated with UI_*i*_. As for RIHT and our scheme, we only need to get the log table's index stored on the request packet's marking field. With the index, we are able to retrieve the logged data from the table without any search. Therefore their computation load is Θ(1). Since RIHT and our proposed scheme do not need to spend time on searching, the path reconstruction in the two schemes is obviously faster than that in MRT and MORE.

**Figure 9:**
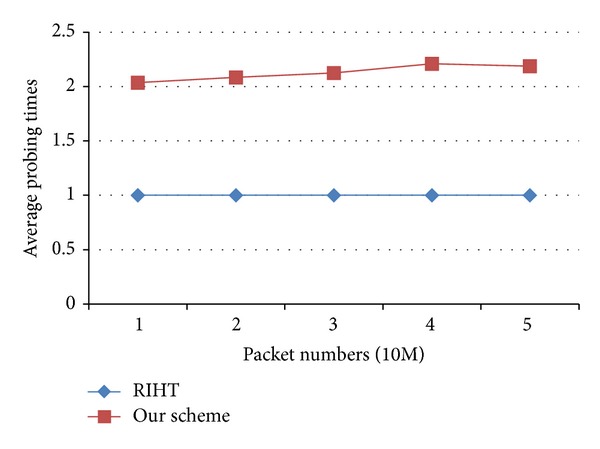
Average probing times in path reconstruction.

Figure 9 demonstrates the relation between packet numbers and average probing times in RIHT and our scheme. Here, average probing times represent the average times of probing in path reconstruction. In our scheme, if there are filled-up tables, we may need more probes to find the exact table where the mark is logged. That is why our average probing times slightly increase with packet numbers; see Figure 9. But, mostly our average probing times are just close to 2. RIHT needs only one search for a logged path because it has only one log table. Following its index, it retrieves the logged data. Our scheme needs at least two searches because we have to find the log table first and then the logged path. The difference of one more search between the two schemes is, in fact, rather insignificant.

### 4.5. False Positive and False Negative Rates

When a router is mistaken as an attack router, we call it a “false positive.” When we fail to trace back to an attacker, we call it a “false negative”. Besides, a router's storage capacity is limited. If packet numbers exceed a router's storage limit, its log tables will be refreshed. Then, false negatives may occur in path reconstruction.

Both MRT and MORE use packet digests as their indexes. Consequently, the size of their log tables grows with the number of logged packets. Figure 8 shows that both MRT and MORE require more storage when packet numbers increase. But a router has only limited storage. When a router runs out of space, the two schemes can only refresh their log tables. And this can cause false negatives. Our scheme requires low storage and does not need to refresh the log tables, so it is able to achieve 0 false positive.

In MRT and MORE, even if their logged data is not cleared, it is still possible for the two schemes to have false positives because of the collision between attacking packets and other packets. The false positive rates for MRT and MORE are *n*/2^32^, and *n*_UI_*i*__/2^32^ respectively, where *n* denotes packet numbers; *n*_UI_*i*__ denotes the number of packets that pass through UI_*i*_; and “32” denotes the number of bits of a packet digest. As a result, we find it obvious that the false positive rates and packet numbers are proportional in MRT and MORE.

**Figure 10:**
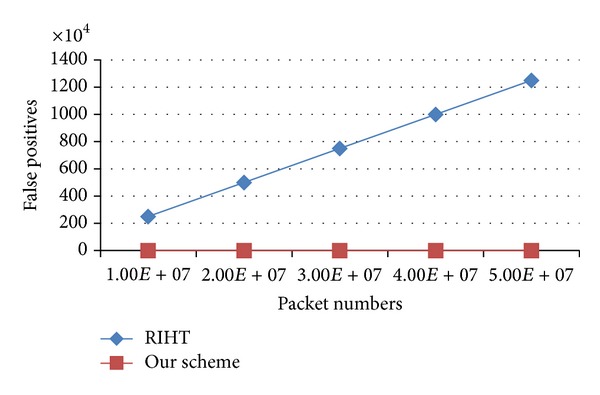
Comparison of false positives.

Unlike the two schemes, RIHT and ours do not use packet digests for indexing. Instead, we use logged packets' other fields to store the log tables' numbers. Therefore, we will not have false positives because of the collision of packet digests. In spite of the claimed 0 false positive in RIHT, it fails to take packet fragmentation into consideration. When a packet is fragmented, the information marked on the packet will be modified. This can cause false positives in path reconstruction. The false positive rate is equal to the fragmentation rate, that is, 0.25%. In our method, we use only a 16-bit ID field for marking. Fragmentation will not cause any change to the field. For this reason, we can say our scheme can truly make 0 false positive in path reconstruction. As shown in Figure 10, RIHT's false positives increase with packet numbers, but ours remains 0.

## 5. Conclusion

In this paper, we propose a new hybrid single-packet traceback scheme that uses only 16 bits for marking. Compared with RIHT, our storage requirement is only 1.5 MB higher and we just need one more search in path reconstruction. It can be seen as practical as RIHT in storage requirement. With only 2 MB storage requirement, the chance of a router refreshing our log tables is quite low. However, RIHT uses 32-bit fields for marking and inevitably suffer from packet dropping if packets are fragmented. Its false positive rate rises with packet numbers. As the simulation results indicate, if compared with the 16-bit hybrid traceback scheme MORE, our scheme requires low storage and low logging times. Among current traceback schemes, ours is the first one whose storage, computation loads, and track accuracy are not affected by packet numbers. Therefore, we can achieve 0 false positive in tracking the origin of attacks with spoofed IPs. In conclusion, our scheme has the best performance in storage and traceback among current 16-bit hybrid IP traceback schemes.

## Figures and Tables

**Algorithm 1:**
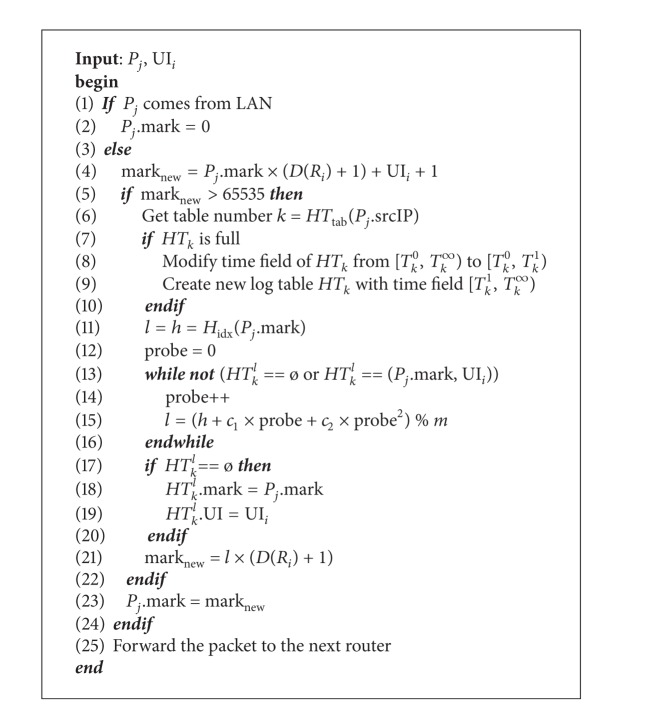
Marking and logging scheme.

**Algorithm 2:**
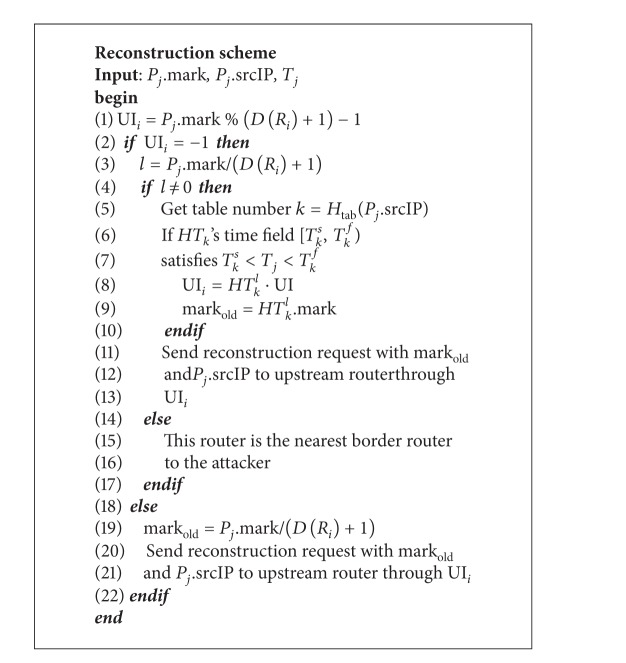
Path reconstruction scheme.

**Table 1:** IP header; Identification field is used for our packet marking.

Bit offset	0–3	4–7	8–15	16–18	19–31
0	Version	Header length	TOS	Total length	
32	**Identification field**			Flag	Fragment offset
64	TTL		Protocol	Header checksum	
96	Source address				
128	Destination address				
160	Options				
160 or 196+	Payload (first 8 bytes)				

**Table 2:** Log table *HT*_*k*_ created at *T*_*k*_^*s*^, full at *T*_*k*_^*f*^.

[*T*_*k*_^*s*^, *T*_*k*_^*f*^)		
*HT*_*k*_		
Index	Mark	UI
0	Source router	
*∙*	*∙*	*∙*
*∙*	*∙*	*∙*
*∙*	*∙*	*∙*
*l*	*P*_*j*_.mark	UI_*i*_
*∙*	*∙*	*∙*
*∙*	*∙*	*∙*
*∙*	*∙*	*∙*

## Notations

*R*_*i*_: {*R*_1_, *R*_2_,…, *R*_*i*_,…, *R*_*x*_}, routers in a network
*D*(*R*_*i*_): The degree of *R*_*i*_
*P*_*j*_: Received packet *P*_*j*_
UI_*i*_: The upstream interface number of router *R*_*i*_
*P*_*j*_.mark: Marking field of *P*_*j*_
*P*_*j*_.srcIP: *P*_*j*_'s source IP
*m*: A log table with *m* entries
*N*: *n* denotes the number of log tables
*c*_1_, *c*_2_: Constants
*H*_tab_(): A hash function with hashed value ranging from 0 to *n* − 1
*H*_idx_(): A hash function with hashed value ranging from 0 to *m* − 1
*HT*_*k*_: Log table *k*
*HT*_*k*_^*l*^: *l*th entry in log table *HT*_*k*_, where *l* ranging from 0 to *m* − 1
[*T*_*k*_^*s*^, *T*_*k*_^*f*^): *T*_*k*_^*s*^ denotes log table *k*'s created time; and *T*_*k*_^*f*^ denotes *k*'s full time, where *s*, *f* = 0 …, *t*,…*∞*. If *s* = 0, it means the first table of *k*. If *f* = *∞*, it means the table has not been filled up
*T*_*j*_: The time that packet *P*_*j*_ arrives at the destination
%: Modulo operation.

## References

[1] Hussain A, Heidemann J, Papadopoulos C A Framework for classifying denial of service attacks.

[2] Yaar A, Perrig A, Song D FIT: fast internet traceback.

[3] Song DX, Perrig A Advanced and authenticated marking schemes for IP traceback.

[4] Tian HC, Bi J, Jiang X-K, Zhang W A probabilistic marking scheme for fast traceback.

[5] Liu JS, Lee Z-J, Chung Y-C (2007). Dynamic probabilistic packet marking for efficient IP traceback. *Computer Networks*.

[6] Savage S, Wetherall D, Karlin A, Anderson T (2001). Network support for IP traceback. *IEEE/ACM Transactions on Networking*.

[7] Paruchuri V, Durresi A, Chellappan S TTL based packet marking for IP traceback.

[8] Belenky A, Ansari N Accommodating fragmentation in deterministic packet marking for IP traceback.

[9] Belenky A, Ansari N (2003). IP traceback with deterministic packet marking. *IEEE Communications Letters*.

[10] Belenky A, Ansari N Tracing multiple attackers with deterministic packet marking (DPM).

[11] Snoeren AC, Partridge C, Sanchez LA (2002). Single-packet IP traceback. *IEEE/ACM Transactions on Networking*.

[12] Bloom BH (1970). Space/time trade-offs in hash coding with allowable errors. *Communications of the ACM*.

[13] Zhang L, Guan Y TOPO: a topology-aware single packet attack traceback scheme.

[14] Gong C, Sarac K (2008). A more practical approach for single-packet IP traceback using packet logging and marking. *IEEE Transactions on Parallel and Distributed Systems*.

[15] Choi KH, Dai HK A marking scheme using Huffman codes for IP traceback.

[16] Malliga S, Tamilarasi A (2010). A hybrid scheme using packet marking and logging for IP traceback. *International Journal of Internet Protocol Technology*.

[17] Malliga S, Tamilarasi A (2008). A proposal for new marking scheme with its performance evaluation for IP traceback. *WSEAS Transactions on Computer Research*.

[18] Yang M-H, Yang M-C (2012). RIHT: a novel hybrid IP traceback scheme. *IEEE Transactions on Information Forensics and Security*.

[19] CAIDA CAIDA’s skitter project. http://www.caida.org/tools/skitter/.

[20] John W, Tafvelln S Analysis of internet backbone traffic and header anomalies observed.

[21] Security Assessment of the Internet Protocol.

[22] Knuth DE (1998). *The Art of Computer Programming, Volume 3: Sorting and Searching*.

